# Gray matter reserve determines glymphatic system function in young‐onset Alzheimer's disease: Evidenced by DTI‐ALPS and compared with age‐matched controls

**DOI:** 10.1111/pcn.13557

**Published:** 2023-05-21

**Authors:** Hsin‐I Chang, Chi‐Wei Huang, Shih‐Wei Hsu, Shu‐Hua Huang, Kun‐Ju Lin, Tsung‐Ying Ho, Mi‐Chia Ma, Wen‐Chiu Hsiao, Chiung‐Chih Chang

**Affiliations:** ^1^ Department of Neurology, Cognition and Aging Center, Institute for Translational Research in Biomedicine, Kaohsiung Chang Gung Memorial Hospital Chang Gung University College of Medicine Kaohsiung Taiwan; ^2^ Department of Radiology, Kaohsiung Chang Gung Memorial Hospital Chang Gung University College of Medicine Kaohsiung Taiwan; ^3^ Department of Nuclear Medicine, Kaohsiung Chang Gung Memorial Hospital Chang Gung University College of Medicine Kaohsiung Taiwan; ^4^ Department of Nuclear Medicine, Lin‐Ko Chang Gung Memorial Hospital Chang Gung University Taoyuan Taiwan; ^5^ Department of Statistics and Institute of Data Science National Cheng Kung University Tainan Taiwan

**Keywords:** diffusion tensor imaging along the perivascular space, glymphatic system, young‐onset Alzheimer's disease

## Abstract

**Background:**

The diffusion tensor imaging analysis along the perivascular space (ALPS)‐index can be used to model the glymphatic system *in vivo*.

**Aim:**

This study explores putative mechanisms between prediction of ALPS‐index and cognitive outcomes in young‐onset Alzheimer's disease (YOAD) and age‐matched controls (CTLs) and analyzes whether the link was mediated by the integrity of ALPS‐index‐anchored cerebral gray matter (GM).

**Methods:**

We enrolled 130 patients with YOAD and 137 CTLs. All participants underwent three‐dimensional T_1_‐weighted MRI, diffusion tensor imaging and cognitive tests. We constructed GM regions correlated with the ALPS‐index in the YOAD and CTL groups. For the GM regions significantly correlated with the ALPS‐index and cognitive measures, we extracted a 4‐mm radius sphere. In the YOAD and CTL groups, we used mediator analysis to explore the ALPS‐index as predictor, GM partitions as mediators, and significant cognitive test scores as outcomes.

**Results:**

Patient group had significantly lower ALPS‐index. The ALPS‐index was associated with GM volume in the cerebellar gray, dorsolateral prefrontal, thalamus, superior frontal, amygdala and hippocampus, and these coherent regions coincided with those showing GM atrophy in the YOAD group. Mediation analysis of the YOAD group suggested that the relationships between the ALPS‐index and cognitive performance were fully mediated by the integrity of ALPS‐index coherent GM areas.

**Discussion:**

Reserved GM mediates the link between the glymphatic system and cognition. Our findings suggest that GM integrity rather than the glymphatic system could serve as a direct cognitive test scores predictor in patients with YOAD.

The glymphatic system is a highly organized fluid‐clearance pathway^1^, ^2^ involving the movement of cerebrospinal fluid (CSF) alongside the perivascular space.^3^ This glymphatic system allows the clearance of extracellular proteins such as amyloid β and tau, while deletion of the AQP4 gene suppresses this clearance capability. The glymphatic system has been visualized *in vivo* using two‐photon imaging with a fluorescent tracer and laser‐scanning microscope.^4^ In humans, direct visualization of the glymphatic flow system has been achieved using MRI tracer studies with intrathecal or intravenous injections of gadolinium‐based contrast agents.^5^ However, the glymphatic system is still a hypothesis, and it involves function rather than an obvious anatomical structure.

In 2017, Taoka *et al*. developed a non‐invasive diffusion tensor image‐based method to measure diffusivity along the perivascular space (ALPS).^6^ On a transverse slice at the level of the lateral ventricle body, they reported significant inverse relationships between diffusivities orthogonal to the projection and association fibers in patients with Alzheimer's disease (AD). The decrease in parameters was speculated to represent decreased diffusivity along the perivascular space, as the medullar veins run perpendicular to the ventricular wall. While the ALPS‐index measures diffusivity in the direction of the perivascular space in the periventricular white matter, it has been proposed to be an indirect indicator of the state of glymphatic function.^6^ As the ALPS‐index has also been positively correlated with Mini‐Mental State Examination (MMSE) score, the integrity of glymphatic function has been linked to the prediction of cognition. In a subsequent study of AD patients,^7^ the ALPS‐index was found to be correlated with cerebral spinal fluid amyloid β, fluorodeoxyglucose positron emission tomography (PET) signals, and cognitive test scores. These findings suggest the glymphatic clearance of amyloid and a relationship with cognitive outcomes in AD. Although the glymphatic hypothesis remains to be established, it is considered to serve important roles in aggregation‐associated diseases including AD,^8^, ^9^, ^10^ Parkinson's disease,^11^, ^12^, ^13^ and sleep disorders.^14^, ^15^

In 2018, the NIA‐AA working group proposed the latest biomarker‐based framework using amyloid and tau pathology^16^ in AD, however glymphatic dysfunction was not included in the consensus. For AD, the framework is amyloid‐centric, followed by a subsequent pathological change in tau that contributes to neuronal injuries such as gray matter (GM) atrophy and clinical syndromes. Despite progress in research in AD, mechanisms for the decreased glymphatic function and cognitive impairment in AD have not been fully explored. If the clinical significance of glymphatic function to AD pathogenesis is at a high hierarchy, the link between the ALPS‐index and cognition should be direct and not mediated by other parameters.

A subset of patients may develop AD before 65 years of age, termed young‐onset AD (YOAD). YOAD accounts for about 5–6% of all cases of AD, and it differs substantially from late‐onset AD in terms of clinical phenotype, genetic predisposition, neuropathological burden, and topography.^17^, ^18^ The genetic predisposition to YOAD involves amyloid precursor protein, presenilin 1 and presenilin 2 mutations,^19^ which may lead to the overproduction of amyloid plaque. The association between the ALPS‐index and YOAD is still unclear, and more evidence is needed to examine whether glymphatic dysfunction also determines cognitive test scores in patients with YOAD.

Based on the glymphatic hypothesis and the use of the ALPS‐index to assess glymphatic function, we explored three research questions in this study. First, whether the ALPS‐index may serve as a putative cognitive predictor in patients with YOAD or age‐matched controls (CTLs). Based on the literature, we hypothesized a lower ALPS‐index in the patients. Second, whether a glymphatic system‐coherent GM network may exist in YOAD. Likewise in age‐matched CTLs, we investigated the spatial extent of glymphatic system‐related GM networks and explored whether there was any overlapping anatomy with YOAD. Third, whether the ALPS‐index plays an independent and direct role on cognitive outcome prediction in patients with YOAD or in CTLs.

## Methods

### Patient enrollment

This was a cross sectional case control study. This study was conducted in accordance with the Declaration of Helsinki and was approved by the Institutional Review Board of Chang Gung Memorial Hospital. The study participants were consequently enrolled and treated at the Cognition and Aging Center, Department of General Neurology, Kaohsiung Chang Gung Memorial Hospital since 2009.

AD was diagnosed by a multidisciplinary team composed of behavioral neurologists, psychiatrists, neuropsychologists, neuroradiologists, and experts in nuclear medicine. We enrolled patients with YOAD who were under 65 years of age at disease onset. The clinical diagnosis was based on the International Working Group‐2 criteria,^20^ verbal episodic memory deficits on the Chinese version of the Verbal Learning Test (CVLT),^21^ and further confirmed by positive amyloid imaging results by two independent raters^22^ and the Centiloid score. The amyloid tracers used in this study included Florbetapir and Florbetaben and the cutoff off value of amyloid Centiloid scores was 40.

Unrelated healthy CTLs aged between 46 and 66 years were recruited from the community. The cognitively unimpaired CTLs had no intracranial disorders, and they all had cognitive test scores above educational‐based cutoff criteria.^21^

The exclusion criteria were a history of stroke, presence of small vessel disease, a negative amyloid scan, depression, or chronic insomnia. After checking the inclusion and exclusion, a total of 130 patients with YOAD (52 males and 78 females) and 137 cognitively unimpaired age matched CTLs (60 males and 77 females) were enrolled for the following study workflows.

### Demographic registration and cognitive assessment

The neurobehavioral tests included the MMSE and Cognitive Abilities Screening Instrument (CASI).^23^ The CASI contains nine subdomains: mental manipulation, attention, orientation, long‐term and short‐term memory, abstract thinking, drawing ability, verbal fluency and language. The subdomains used to assess executive function include attention, verbal fluency, abstract thinking, and mental manipulation subdomain scores.^23^ We considered orientation, short‐term and long‐term memory, language ability, and drawing to be non‐executive domains.

After enrollment, blood samples were collected and the following were measured: (1) cerebral vascular risk factors: homocysteine, high sensitivity C‐reactive protein, total cholesterol, triglycerides, high‐density lipoprotein, low‐density lipoprotein, very low‐density lipoprotein, (2) metabolic and nutritional factors: fasting glucose, glycohemoglobin (HbA1c), calcium, blood urea nitrogen, creatinine, aspartate aminotransferase, alanine aminotransferase, cortisol, triiodothyronine, thyroxine, thyroid stimulating hormone, cobalamin (B12), folate and albumin. The apolipoprotein 4 (*APOE4*) genotype was determined using rs7412 and rs429358. *APOE4* carriers were defined as those with one or two E4 alleles.^24^

### Image acquisition

All images were acquired using a 3.0 Tesla scanner (Skyra, Siemens Medical Systems, Erlangen, Germany) with a 12‐channel head coil. High‐resolution T1‐weighted anatomical images were obtained using a fast spoiled gradient echo sequence with the following parameters: TR 2600 msec, echo time 3.15 msec, flip angle 13°, 176 slices with sagittal acquisition, slice thickness 1 mm, and voxel size 1 × 0.5 × 0.5 mm^3^.

The diffusion‐weighted images were acquired using a single‐shot spin‐echo echo‐planar‐imaging sequence. Images of *b* = 1000 s/mm^2^ were acquired with diffusion‐weighted gradients applied along 64 non‐collinear directions. Axial images were acquired using the following parameters: TR/TE 8800/91 ms; 70 axial slices, flip angle 90°, slice thickness 2.2 mm; field of view 255 × 255 mm^2^. The total image acquisition time was 40 min.

### DTI‐ALPS processing

Preprocessing of the diffusion‐tensor images included the following steps: (1) denoising using Marchenko‐Pastur principal component analysis^25^; (2) removal of Gibbs ringing^26^; (3) correction of motion and distortion artifacts^27^, ^28^; and (4) correction of bias field. The bias field was first estimated from *b* = 0 in diffusion‐weighted imaging data and then applied to correct all diffusion‐weighted imaging volumes.^29^

Diffusivity maps in the direction of the x‐axis (Dxx), y‐axis (Dyy) and z‐axis (Dzz) and color‐coded fractional anisotropy (FA) were generated by MRtrix3 (version 0.3.15).^30^ In this study, the corticofugal corona radiata projection represented the projection fibers, and the superior longitudinal fasciculus represented the association fibers to calculate the ALPS‐index.^6^, ^31^ Based on the JHU atlas (ICBM labels 2mm),^32^ we created 5‐mm thickness masks at the lateral ventricle body level in projection fiber and association fiber regions. The regions of interests were placed on both sides, and the values from both sides were averaged.

Both the transformed matrices between the Montreal Neurological Institute (MNI)‐152 spaces and individual 3D T1WI images, as well as 3D T1WI and FA images were obtained. The concatenated transformation matrix was obtained to transform the MNI‐152 spaces (JHU atlas masks) into FA native spaces. Using the concatenated transformation matrix, we recorded diffusivity values in the directions of the x‐axis, y‐axis and z‐axis on the projection fibers and association fibers as Dxxproj, Dyyproj, Dzzproj, Dxxassoc, Dyyassoc and Dzzassoc. The ALPS‐index was calculated as [mean (Dxxproj+Dxxassoc)/mean (Dyyproj+Dzzassoc)].

### High‐resolution T1 volumetric processing

Individual 3D T1 image preprocessing and statistical analysis were performed using SPM12 (Wellcome Trust Centre of Cognitive Neurology, University College London, UK, http://www.fil.ion.ucl.ac.uk/spm/) and its extension toolbox (CAT12, https://neuro-jena.github.io/cat/). The T1 images were reoriented, re‐aligned and normalized using the standard Montreal Neurological Institute (MNI) template. The segmentation of tissue type was performed using partial volume estimation.^33^ The modulated and warped GM images were then smoothed using a Gaussian kernel of 8 mm full width at half maximum.^34^

### Statistical analysis

Clinical data were expressed as mean and standard deviation (SD). We tested data normality using Shapiro–Wilk test. The Student's t test was used to compare cognitive test scores between the YOAD and CTL groups. Correlation analysis was performed using Spearman's correlation analysis, adjusted for possible confounders as detailed. Statistical significance was set at *P* < 0.05. All statistical analyses were performed using R software version 4.2.1.

To detect YOAD‐related cortical atrophy, voxel‐based statistics were performed by comparing differences in modulated GM images with the CTLs. The results were thresholded at a family‐wise error of *P*< 0.05 at the voxel level for multiple comparisons and a cluster threshold of 100 voxels.

To understand whether the ALPS‐index serve as a putative cognitive biomarker in the patients with YOAD or CTL, we reported partial correlation analysis and multivariate regression analysis results using ALPS‐index as dependent variable and the cognitive test score, age and educational years as independent variables.

Further, in both YOAD and CTL groups we performed general linear regression analysis between the ALPS‐index and smoothed modulated GM, adjusted for estimated total intracranial volume (eTIV). Significance was set using an uncorrected threshold of *P* < 0.001 at the cluster level and a cluster size >100 voxels. The statistical model helped to establish the ALPS‐index coherent GM regions in the CTL and YOAD groups.

To understand whether the ALPS‐index can serve as a direct cognitive marker of YOAD or whether its relationship with cognition is mediated by another indirect pathway such as GM integrity, we further extracted GM partition volumes as mediator. For GM regions showing statistical significance with ALPS index, the peak MNI coordinates were first recorded and double checked the location on the AAL3 template for anatomical labelling. A 4‐mm radius sphere mask was generated in template space and transferred back to the native subject space *via* the inverse transformation matrix. A 4‐mm radius sphere was chosen because a spherical mask of this size could cover the anatomical structure we intended to investigate. As the pathology in typical patients with AD is distributed symmetrically, we only performed seed analysis in the left hemisphere. The seed volume represented the mediator variable for the mediation analysis.

The mediation analysis was performed using the Preacher and Hayes mediation method,^35^ using the ALPS‐index as the predictor, predefined GM regions of interest as the mediators, and cognitive test scores as the outcomes. We used bootstrapping tests with 1000 resamples and the bias‐corrected confidence interval. In our model, the total direct pathway represented the ALPS‐index to cognition, and the indirect pathway represented the ALPS‐index ‐ > GM partition ‐ > cognition. For the YOAD group, we used CASI total score and short‐term memory score as the cognitive outcomes. Likewise, in the CTLs, the mediation analysis was performed in significant GM regions, and the cognitive test scores were correlated with the ALPS‐index. Significance was set at *P* < 0.05.

### Standard protocol approvals, registrations, and patient consents

This study was approved by the Institutional Review Board of Chang Gung Memorial Hospital. Written informed consent was obtained from all participants. The study approval numbers were 202001125B0, 201801829B0A3 and 201800911A3.

## Results

### Demographic data and the ALPS‐index

The demographic data of the YOAD and CTL groups are shown in Table 1. The amyloid Centiloid score of YOAD group was 68 ± 32.1. The proportion of *APOE*4 carriers was higher in the YOAD group (23.8%) compared with the CTL group (11.4%, *P* = 0.037). While there was no significant difference in sex between the two groups, the CTL group had more years of education. Compared with the CTL group, the YOAD group had significantly lower scores in all cognitive tests, lower B12 levels, and higher blood glucose and HbA1c levels (Table S1).

**Table 1:** Demographic data comparisons

	Diagnosis	Mean	SD	*P* value
Female	CTL	77		0.53
	YOAD	78		
Educational years	CTL	12.47	3.63	3.96 × 10^−8^
	YOAD	9.59	4.38	
Age at image study (year‐old)	CTL	58.52	5.71	0.12
	YOAD	60.15	3.67	
Mini‐Mental State Examination (30)	CTL	28.01	1.80	1.22 × 10^−30^
	YOAD	19.55	7.77	
CASI Total Score (100)	CTL	92.41	5.21	2.18 × 10^−24^
	YOAD	65.97	26.91	
Mental Manipulation (10)	CTL	8.98	1.36	9.10 × 10^−11^
	YOAD	5.88	3.74	
Attention (8)	CTL	7.50	0.68	5.14 × 10^−10^
	YOAD	6.07	2.08	
Orientation (18)	CTL	17.61	1.03	1.62 × 10^−15^
	YOAD	12.82	5.80	
Long term memory (10)	CTL	9.89	0.46	3.38 × 10^−8^
	YOAD	7.84	3.52	
Short term memory (12)	CTL	10.86	1.38	2.65 × 10^−24^
	YOAD	5.35	3.88	
Abstract thinking (12)	CTL	10.12	1.68	1.08 × 10^−10^
	YOAD	7.49	3.38	
Drawing ability (10)	CTL	9.63	1.14	1.93 × 10^−11^
	YOAD	7.50	3.17	
Verbal fluency (10)	CTL	8.08	1.83	1.48 × 10^−13^
	YOAD	5.09	3.11	
Language (10)	CTL	9.75	0.48	8.50 × 10^−9^
	YOAD	7.97	2.87	

Note: Numbers in the parenthesis following cognitive tests indicate maximal score.

Abbreviation: YOAD: Young onset Alzheimer's disease (*n* = 130); CTL: age‐matched controls (*n* = 137). CASI, Cognitive Ability Screening Instrument; SD, standard deviation.

### Lower ALPS‐index in the YOAD group

**Fig. 1:**
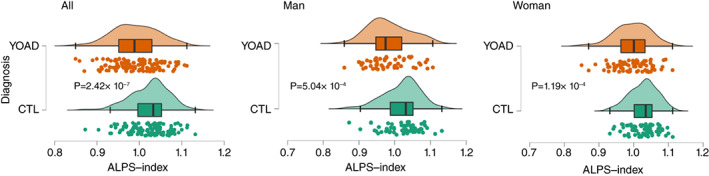
Distribution of the ALPS‐index in all participants (All), male or female controls (CTLs) and patients with young‐onset Alzheimer's disease (YOAD). ALPS, diffusion tensor imaging along the perivascular space; *P*, *P*‐value.

The YOAD group had a significantly lower ALPS‐index than the CTL group (Fig. 1) in both genders. In the patients with YOAD, the *APOE*4 carriers were not associated with a lower ALPS‐index than the non‐carriers (*P* = 0.385). In the CTLs or YOAD (Figure S1), the correlations between the ALPS‐index and age and ALPS‐index and education years were not significant.

### ALPS‐index predicted cognitive scores

In both the YOAD and CTL groups, there were significant correlations between the ALPS‐index and cognitive outcomes, however, the significant subdomains were different. In the YOAD group (Table 2, Table S2), positive correlations between the ALPS‐index and MMSE, CASI, short‐term memory, drawing ability and language scores were found even after adjusting for age and education years. Of specific notes, the significant CASI subdomains were in the non‐executive categories. In the CTL group (Table S2), positive correlations between the ALPS‐index and mental manipulation, long‐term memory and short‐term memory scores were found, however only the subdomain of mental manipulation remained significant after adjusting for age and education years.

**Table 2:** Regression relationships between cognitive tests and ALPS‐index in the patients

Cognitive test	Unstandardized beta	95% confidence interval (lower–upper)	*P*‐value
Mini‐mental state examination	30.689	6.419–54.958	0.014
CASI total (100)	94.463	5.404–183.522	0.039
Mental manipulation (10)	9.742	−2.398–21.883	0.115
Attention (8)	5.895	−1.204–12.994	0.103
Orientation (18)	17.460	−2.126–37.045	0.121
Long term memory (10)	8.484	−3.236–20.205	0.154
Short term memory (12)	15.703	2.635–28.770	0.019
Abstract thinking (12)	7.145	−4.240–18.530	0.216
Drawing ability (10)	10.438	0.363–20.512	0.042
Verbal fluency (10)	9.470	−1.090–20.031	0.078
Language (10)	10.155	0.705–19.606	0.035

*Note*: Dependent variable: Cognitive test score. Independent variables: ALPS‐index, education years and age.

Abbreviation: ALPS, diffusion tensor imaging along the perivascular space; CASI, Cognitive ability screening instrument, numbers in the parenthesis indicate maximal values.

Among the blood profiles, the ALPS‐index was inversely related to hemoglobin levels in the YOAD group (Table S3).

### GM atrophy map in the patients with YOAD overlapped considerably with the ALPS‐index‐anchored GM regions

**Fig. 2:**
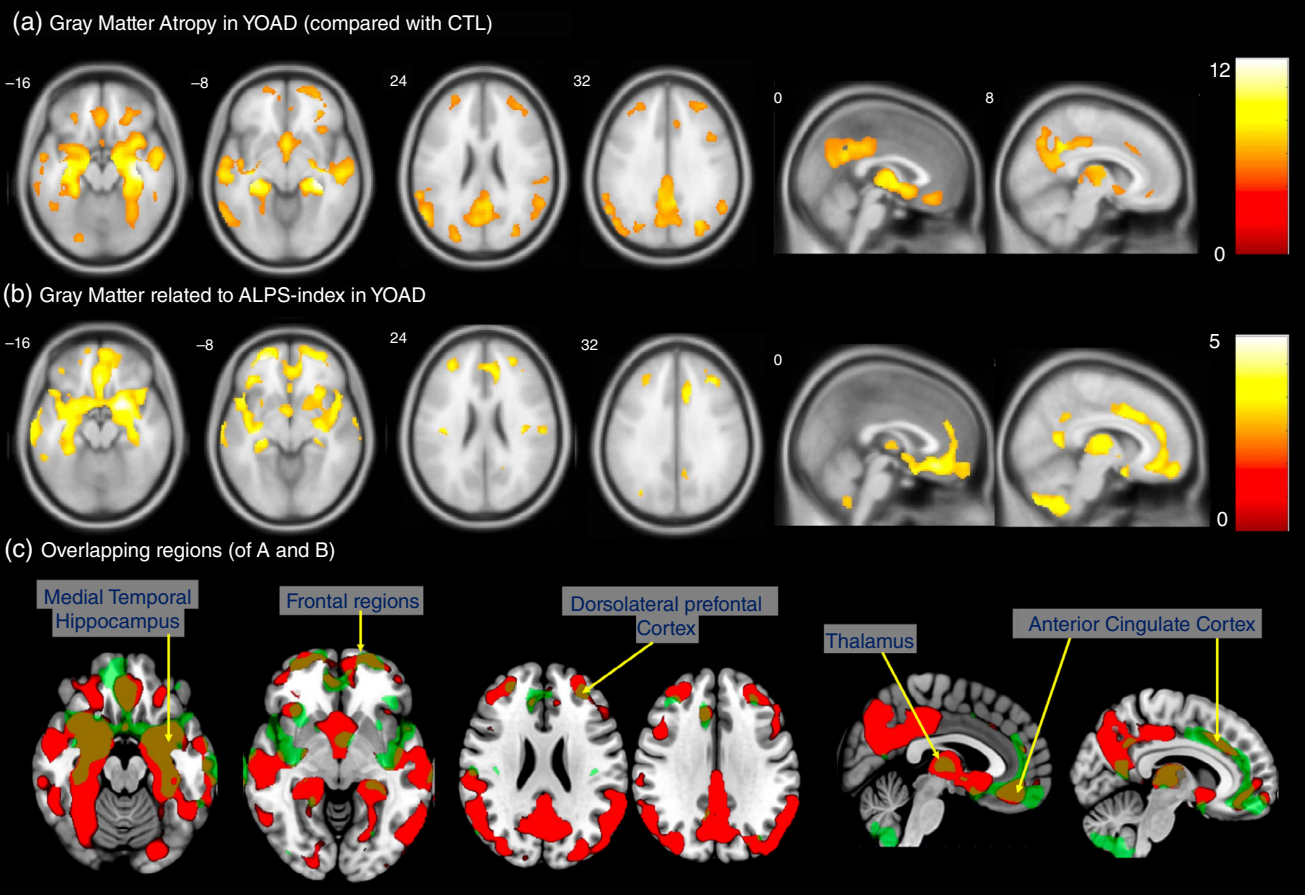
(A) Voxel‐based comparisons of patients with young‐onset Alzheimer's disease (YOAD) with age‐matched controls. *P* < 0.05, correlated for multiple comparisons using family‐wise error. (B) Gray matter regions correlated with the ALPS‐index in the patients with YOAD with a statistic threshold of *P*_uncorrected_ < 0.001 and cluster size >100 voxels. (C) Regions showing overlapping in (A) and (B) in brown. Color bar represents *T* statistics. ALPS, diffusion tensor imaging along the perivascular space.

Compared with the CTLs, the patients with YOAD showed GM atrophy in the hippocampus, precuneus, temporal–parietal junction, anterior and middle cingulate, lateral frontal and thalamus regions (Fig. 2A, Table S4). GM regions associated with the ALPS‐index, adjusted for eTIV, were in the hippocampus, anterior cingulate, dorsolateral prefrontal, thalamus and cerebellar areas (Fig. 2B, Table S5: YOAD). The topography showing GM atrophy and ALPS‐coherent regions in the YOAD group shared overlapping clusters in the hippocampus, dorsolateral prefrontal, frontal, thalamus and anterior cingulate areas (Fig. 2C Brown color).

### Mediation analysis in the patients with YOAD showed full mediation of GM partitions in ALPS‐index and cognition

**Fig. 3:**
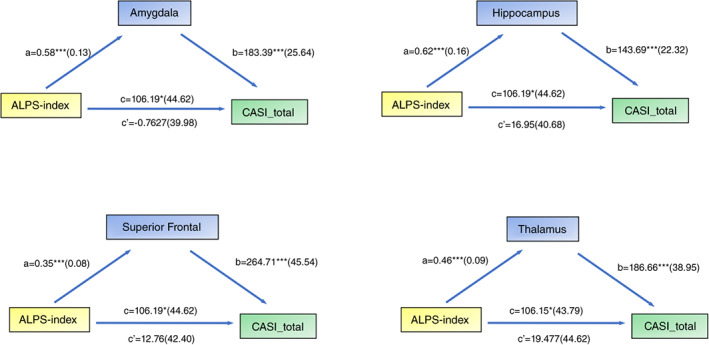
Simple mediation diagram in YOAD; a, b, c, and c' are path coefficients representing unstandardized regression weights and standard errors (in parentheses). The c path coefficient represents the total effect of the ALPS‐index on CASI total scores. The c' path coefficient refers to the direct effect of the ALPS‐index on CASI total scores. All analyzed a, b, and c paths were significant, *P < 0.05, ***P < 0.001. ALPS, diffusion tensor imaging along the perivascular space; CASI, Cognitive Ability Screening Instrument. YOAD, young‐onset Alzheimer's disease.

As significant correlations between the ALPS‐index and cognitive outcomes were established, we tested whether this relationship was mediated by ALPS‐index coherent GM clusters. The significant GM clusters related to MMSE and ALPS‐index is shown in Table 3. Mediation analysis was performed using the ALPS‐index as the predictor, GM partitions of amygdala, hippocampus, superior frontal and thalamus as the mediators, and cognitive test scores as the outcomes. The results showed that both the CASI total score (Fig. 3) and short‐term memory test score (Figure S2) were affected by the ALPS‐index *via* the full mediation of predefined GM clusters.

**Table 3:** Gray matter clusters related to MMSE and ALPS‐index in YOAD

Cluster	Cluster size	Anatomy & lateralization		*X*	*Y*	*Z*	*T*	*P*
I	3469	Cerebellum	R	15	−69	−54	5.15	<0.0001
II	1929	Amygdala	R	21	6	−16	4.72	<0.0001
		Hippocampus	R	32	−14	−12	4.12	<0.0001
III	2533	Cerebellum	L	−24	−70	−54	4.68	<0.0001
		Cerebellum crus	L	−36	−70	−46	3.99	<0.0001
IV	498	Superior frontal	L	−26	54	−8	4.66	<0.0001
V	1310	Thalamus	L	−20	−30	3	4.51	<0.0001
VI	1058	Thalamus	R	18	−30	3	4.5	<0.0001
VII	189	Superior temporal pole	L	−33	18	−28	4.09	<0.0001
VIII	302	Middle cingulate	R	14	16	39	3.92	<0.0001
IX	420	Middle frontal	R	27	52	−9	3.9	<0.0001
		Superior frontal	R	21	58	−2	3.84	<0.0001
X	351	Rectus	L	0	30	−15	3.88	<0.0001
XI	125	Insula	R	48	6	−3	3.77	<0.0001
XII	160	Insula	L	−40	9	0	3.7	<0.0001
XIII	130	Calcarine	R	9	−58	12	3.53	<0.0001

*Note*: Data adjusted estimated total intracranial volume. *X*, *Y*, *Z* represent MNI coordinate; the anatomy is based on the AAL3 template. *P* uncorrected, with cluster size >100 voxels.

Abbreviation: ALPS, diffusion tensor imaging along the perivascular space; CTL, age‐matched controls; L: left; MMSE, Mini‐Mental State Examination; R, right; YOAD, Young onset Alzheimer's disease.

### ALPS‐index coherent GM clusters in the CTLs

**Fig. 4:**
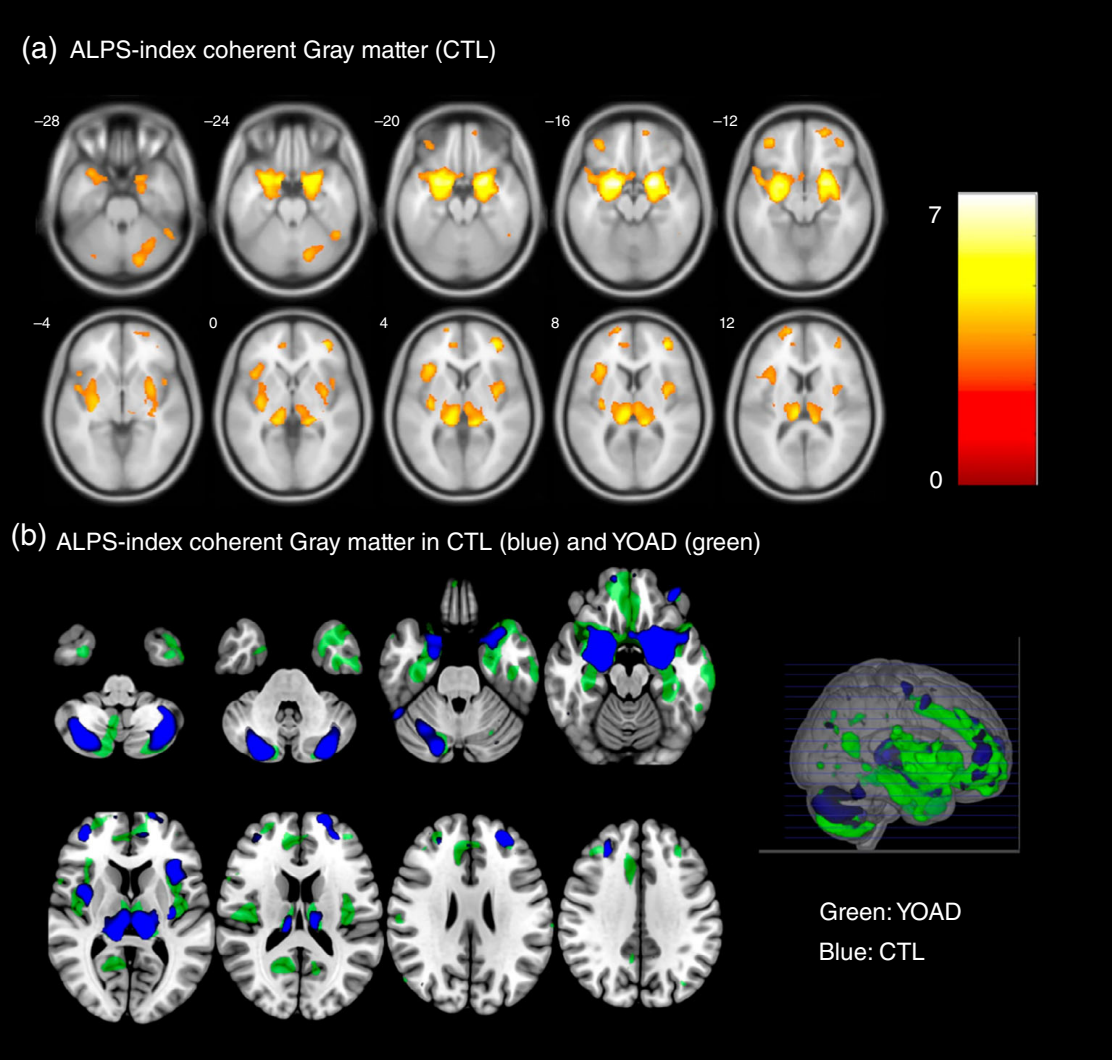
(A) Gray matter regions correlated with the ALPS‐index in age‐matched controls (CTLs) with a statistic threshold of *P*_uncorrected_ <0.001 and cluster size >100 voxels. Color bar represents T statistics. (B) Topography of ALPS‐index coherent gray matter in the CTLs (blue) and patients with YOAD (green). ALPS, diffusion tensor imaging along the perivascular space; YOAD, young‐onset Alzheimer's disease.

In the CTLs, we further explored GM structures that were related to the ALPS‐index, adjusted for eTIV (Fig. 4A, Table S5: CTL cluster). These regions included insula, amygdala‐hippocampus, thalamus, dorsolateral prefrontal regions, and cerebellar GM. In comparisons of topography between the CTL and YOAD groups in ALPS‐coherent regions (Fig. 4B: green: YOAD, blue: CTL), the overlapping regions included cerebellar gray, amygdala‐hippocampus, thalamus, prefrontal and dorsolateral prefrontal. Meanwhile, the ALPS‐index coherent GM in the YOAD group was spatially larger than that shown in the CTL group.

### Mediation analysis in the CTLs showed full mediation of amygdala partitions in ALPS‐index and cognition

GM regions related to the ALPS‐index and mental manipulation in the CTL group are shown in Table S6 and we explored the mediation of GM partitions of amygdala, subgenual frontal region, insular, pulvinar, dorsolateral prefrontal cortex and hippocampus on mental manipulation scores. We found that the mental manipulation scores in the CTLs were affected by the ALPS‐index *via* full mediation of amygdala regions, while in other regions the mediation effect was not observed (Figure S3).

## Discussion

### Key results

In this study, we used the ALPS‐index to reflect glymphatic function and found it functioned differently in YOAD and CTLs. There were three major findings. First, the ALPS‐index was significantly lower in the patients with YOAD compared to age‐matched CTLs. The patients with YOAD who were *APOE*4 carriers were not associated with a lower ALPS‐index than the non‐carriers. Moreover, the ALPS‐index was related to different cognitive domains in the YOAD and CTL groups. Second, we constructed GM networks that covaried with the ALPS‐index in both the YOAD and CTL groups. The GM spatial extents were larger in the YOAD group and the overlapping regions between the YOAD and CTL groups including medial temporal region, hippocampus, dorsolateral prefrontal, thalamus, and cerebellar GM. Third, we speculate that the clinical significance of the glymphatic system on cognitive outcomes may be *via* mediation of the ALPS‐index coherent GM regions. Our analysis confirmed that the association of the ALPS‐index and cognition was fully mediated by GM reserve in the amygdala, superior frontal, thalamus, and hippocampus in the patients with YOAD. In the CTLs, the amygdala had a full mediation effect between the ALPS‐index and mental manipulation. Taken together, these findings showed that the ALPS‐index in the YOAD group or CTLs was not directly associated with cognitive outcomes, but rather that its influence on cognition was mediated by GM reserve.

### Impaired glymphatic function results in brain parenchymal protein accumulation and GM injury

After intravenous gadolinium injection, enhancement of brain parenchymal blood vessels, perivascular spaces, brain parenchyma, choroid plexus and CSF suggest the small molecules transport system from the blood stream to the brain.^36^ In patients with idiopathic normal‐pressure hydrocephalus in whom glymphatic system clearance was delayed,^37^ enhancement of brain parenchyma following gadolinium injection was observed in the pons, thalamus, periventricular frontal horn, inferior frontal gyrus, and precentral gyrus. Taoka *et al*.^38^ discussed the mechanisms for gadolinium deposition in the brain and reported that the glymphatic system may be involved in the route of gadolinium deposition. The delayed clearance of tracer in these regions supports the glymphatic system theory and possible regional vulnerabilities related to glymphatic clearance dysfunction.

The tau propagation theory^39^ highlights the prion‐like nature of tau, and that the trajectory of GM degeneration is triggered by a specific subset of tau species. In our analysis of the patients with YOAD, the associations of ALPS‐index with cognitive test scores were fully mediated by coherent GM volumes, suggesting that glymphatic function plays a minor or neglectable role in YOAD cognition prediction. The function of glymphatic system relied on the integrity of certain GM areas. As the ALPS‐index coherent GM network was still wider than those in CTLs, it is also possible that increased pathological load outweighs glymphatic compensatory functions in patients with YOAD.

Conflicting evidence on the concept of the glymphatic system theory exists in the literature. Distribution of CSF‐interstitial fluid in brain tissue has been demonstrated to be *via* interstitial flow, with arterial pulsation as the driving force.^1^ However, a mathematical model^40^ suggested that arterial pulsation would only result in diffusion of molecules to the perivascular space rather than triggering interstitial flow. A review article^41^ found that the lymphatic system and CSF have different protein concentrations, and that therefore use of the glymphatic system is not precise. In addition, observations with a two‐photon microscope are different from physiological conditions, as CSF in the perivascular space is almost immovable. Therefore, the putative mechanism of glymphatic system dysfunction in YOAD needs further investigations.

### Protective role of the glymphatic system *via* brain reserve

As a higher ALPS‐index was associated with higher cognitive test scores in both the YOAD and CTL groups, and due to the full mediating effect of ALPS‐index coherent GM clusters, we propose that the protective role of glymphatic function on cognition may be tightly linked with brain reserve.^42^ Brain reserve reflects the quantity of available neuronal substrate and resilience to pathological damage. A certain proportion of older adults with normal cognition harbor intracerebral amyloid deposits, suggesting that compensatory brain reserve may serve as a protective buffer against amyloid toxicity. In patients with mild AD with high global amyloid load and white matter hyperintensities, prefrontal lobe brain reserve may modulate adverse pathological effects.^43^ Of note, our mediation analysis results are not against the protective role of the glymphatic system on cognitive outcomes. Rather, the influence of glymphatic function on cognition was *via* its protective effect on preserving GM integrity.

### Regional vulnerability to glymphatic dysfunction

Two previous studies investigated associations between AD pathological proteins and glymphatic dysfunction. The first^44^ used amyloid and tau PET to assess a group of patients with AD. The results showed that decreased CSF clearance resulted in increased amyloid and tau, and consequently a reduction in cortical thickness and cognitive decline. In the other study,^45^ tau pathology and MRI experiments provided a spatial and temporal description of the glymphatic system in the brains of transgenic mice. Both studies support the glymphatic hypothesis that failed CSF clearance is characteristic of AD. However, the experiments were not able to elucidate whether there was regional vulnerability related to impaired glymphatic function. Our findings of ALPS‐index coherent regions are consistent with the medial temporal regions of the default mode network that represent salient networks in AD.

A previous study reported that one night of sleep deprivation led to detectable increases in amyloid in the hippocampus and thalamus in healthy CTLs using amyloid PET, and that hours of sleep were inversely related to amyloid deposition in bilateral putamen, parahippocampus, and right precuneus.^46^ In that study, dysfunction of the glymphatic system due to sleep deprivation or a chronically reduced amount of sleep was proposed to cause the accumulation of amyloid in certain regions of the brain, suggesting the regional vulnerability of glymphatic dysfunction. However, GM volume changes were not analyzed in the study, as detection of amyloid accumulation by PET was performed twice in a short interval.

### ALPS‐index coherent GM regions in cognitively unimpaired older people reflect vulnerable regions in aging

Recently, Siow *et al*.^14^ assessed the association between regional GM volume and the ALPS‐index in community‐dwelling older people (mean age 73.3 years), and found that medial frontal, thalamus, medial orbital gyrus, posterior insular, temporal pole and hippocampus pertained to ALPS‐index coherent GM regions. Our CTL group, although younger (mean age 58.5 years), also showed the same regions of the hippocampus, temporal pole, thalamus, and medial frontal. The same coherent regions in different age groups may indicate a close interaction between these GM regions and the glymphatic system. As both our CTLs and the participants in Siow *et al.'s* study^14^ were cognitively unimpaired older adults, whether these ALPS‐index coherent GM regions may represent vulnerable regions in aging requires more evidence. Of note, the mediation analysis in our study showed the important effect of GM integrity of the amygdala in the age‐related mental manipulation scores, while the role of the ALPS‐index in mental manipulation scores became insignificant. Again, this finding is not against the importance of the ALPS‐index, as positive relationships between the ALPS‐index and GM partitions were also found in the CTLs, indicating that the two parameters may have parallel or colinear roles relating to cognitive performance in CTLs.

### Limitations

There are several limitations to this study. First, we only measured the ALPS‐index once, and analysis of the interactions among the ALPS‐index, GM, and cognitive outcomes was based on a neuroimaging model and cross‐sectional design. It is beyond the scope of this study to investigate the effect of alterations in the glymphatic system on neurodegeneration in AD. A longitudinal design with repeated MRI measurements would allow for the *in vivo* modeling of glymphatic alterations and brain degeneration areas. If the glymphatic hypothesis is the major pathophysiology of AD, a decrease in the ALPS‐index may be parallel to the trajectories of cortical atrophy and clinical progression.

Second, our study population was in a narrow age range, so an inverse relationship between the ALPS‐index and age was not established in our YOAD group. The relationship between age and ALPS‐index has been repeatedly demonstrated.^14^, ^47^, ^48^, ^49^ In aging CTLs, the ALPS‐index was shown to have an age‐related second‐degree regression distribution which peaked in those aged in their 40s.^50^ As the enrolled CTLs in the current study were age‐matched to the patients with YOAD, the effect of age on a lower ALPS‐index in the YOAD group may be negligible, and the lower ALPS‐index in the patients with YOAD could be explained by a disease effect.

Finally, the analysis was based on the glymphatic hypothesis, and the ALPS‐index was calculated as a global index which cannot reflect regional glymphatic dysfunction. Further studies investigating regional glymphatic function in AD susceptible networks are needed, as the influence of aggregated protein toxicity may not be the same throughout the whole brain.

## Conclusion

In this study, we used the ALPS‐index to investigate the glymphatic system and found that patients with YOAD had a lower ALPS‐index than age‐matched CTLs. GM regions related to the ALPS‐index in the patients with YOAD and CTLs overlapped considerably, and these regions also constituted the atrophic regions in the patients with YOAD. The glymphatic system may exert its role on ALPS‐index‐anchored GM networks to affect the cognitive performance in patients with YOAD or in CTLs. As such, the ALPS‐index may serve as a protective biomarker for brain reserve.

## Disclosure statement

The authors have no conflict of interests to declare that are relevant to the content of this article.

## Author contributions

Hsin‐I Chang: Drafting of the manuscript for content, including medical writing for content; Analysis and interpretation of data. Chi‐Wei Huang: Study design, clinical and image data acquisition; Analysis and interpretation of data. Shih‐Wei Hsu: Acquisition and analysis of MRI data, revision of the manuscript for content. Shu‐Hua Huang, Kun‐Ju Lin, Tsung‐Ying Ho: Acquired and Analysis and Rating of amyloid PET data, revision of the manuscript for content. Mi‐Chia Ma: statistical analysis for mediation analysis, revision of the manuscript for content. Wen‐Chiu Hsiao: Writing review and editing visualization, revision of the manuscript for content. Chiung‐Chih Chang: Study design and concept, supervision and critical revision. All authors have read and agreed to the published version of the manuscript.

## Supporting information


**Table S1.** Blood profiles comparisons.
**Table S2.** Relationships between cognitive tests and ALPS‐index.
**Table S3.** Relationships between blood profiles and ALPS‐index.
**Table S4.** Gray matter Clusters showing atrophy in YOAD.
**Table S5.** Gray matter clusters related to ALPS‐index.
**Table S6.** Gray matter clusters related to mental manipulation and ALPS‐index in controls.

**Figure S1.** Scatter plots between the ALPS‐index and ages (A) and educational years (B) in control group (CTL) and in patients with young‐onset Alzheimer's disease (YOAD) (C: age, D: educational years).

**Figure S2.** Simple mediation diagram in patients with young onset Alzheimer's disease.

**Figure S3.** Simple mediation diagram in controls.

## Data Availability

The data that support the findings of this study are available on request from the corresponding author and the authors take full responsibility for the data, the analyses and interpretation and the conduct of the research.
