# Supplementary material for: Gray matter reserve determines glymphatic system function in young‐onset Alzheimer's disease: Evidenced by DTI‐ALPS and compared with age‐matched controls
Source: Psychiatry Clin Neurosci. 2023 May 21;77(7):401–9. doi: 10.1111/pcn.13557 (PMC11488612; doi:10.1111/pcn.13557)
Supplement: Supplementary file 1 — Table S1. Blood profiles comparisons. Table S2. Relationships between cognitive tests and ALPS‐index. Table S3. Relationships between blood profiles and ALPS‐index. Table S4. Gray matter Clusters showing atrophy in YOAD. Table S5. Gray matter clusters related to ALPS‐index. Table S6. Gray matter clusters related to mental manipulation and ALPS‐index in controls. [file PCN-77-401-s003.docx]

**Gray Matter Reserve Determines Glymphatic System Function in Young-Onset Alzheimer’s Disease: Evidenced by DTI-ALPS and Compared with Age-matched Controls**

**Supplementary Files**

**eTable 1: Blood profiles comparisons**

**eTable 2: Relationships between cognitive tests and ALPS-index**

**eTable 3: Relationships between blood profiles and ALPS-index**

**eTable 4: Gray matter Clusters showing atrophy in YOAD**

**eTable 5: Gray matter clusters related to ALPS-index**

**eTable6:  Gray matter clusters related to mental manipulation and ALPS-index in controls**

**eFigure Legends 1~3**

**eTable 1: Blood profiles comparisons**

|  | Age-matched Controls | YOAD | P value |
| --- | --- | --- | --- |
| Homocysteine, μmole/L | 10.7 (3.76) | 11.7 (4.54) | 0.21 |
| Fasting Glucose | 98.9 (11.82) | 113.6 (38.38) | 0.03^*^ |
| High sensitive C reactive protein (mg/L) | 2.6 (6.27) | 3.0 (8.70) | 0.81 |
| Hemoglobin A1C, % | 5.9 (0.60) | 6.3 (1.18) | 0.02^*^ |
| Blood Urea Nitrogen, mg/dL | 13.9 (3.24) | 15.4 (5.77) | 0.15 |
| Calcium, mg/dL | 9.4 (0.44) | 9.4 (0.46) | 0.80 |
| Creatinine, mg/dL | 0.8 (0.22) | 0.9 (0.28) | 0.16 |
| Aspartate aminotransferase, U/L | 25.9 (11.43) | 25.0 (9.54) | 0.61 |
| Alanine aminotransferase, U/L | 23.6 (15.48) | 24.8 (16.19) | 0.65 |
| Total cholesterol, mg/dL | 197.8 (39.54) | 203.2 (42.50) | 0.18 |
| Triglyceride, mg/dL | 122.1 (61.64) | 144.1 (131.07) | 0.24 |
| High-density lipoprotein, mg/dL | 55.0 (14.03) | 54.8 (14.98) | 0.96 |
| Very low-density lipoprotein, mg/dL | 25.3 (12.33) | 26.7 (14.90) | 0.60 |
| Low-density lipoprotein, mg/dL | 115.2 (36.35) | 121.8 (35.05) | 0.29 |
| Cortisol, mcg/dL | 12.2 (4.57) | 11.6 (4.29) | 0.51 |
| T4, ng/dL | 1.1 (0.20) | 1.1 (0.19) | 0.40 |
| T3, ng/dL | 94.4 (14.79) | 98.7 (18.49) | 0.26 |
| TSH, μIU/mL | 1.8 (1.16) | 2.1 (1.18) | 0.15 |
| Vitamin B12, pg/dL | 980.2 (857.86) | 654.9 (340.03) | 1.40×10^-3 ***^ |
| Folate, ng/dL | 12.4 (6.52) | 10.9 (5.71) | 0.15 |
| Albumin, g/dL | 4.6 (0.25) | 4.6 (0.35) | 0.89 |
| Hemoglobin, g/dL | 13.7 (1.45) | 13.8 (1.22) | 0.59 |

Data present as mean (standard deviation), ^*^ p<0.05, ^***^ p<0.001

YOAD: Young onset Alzheimer’s disease

| **eTable 2: Relationships between cognitive tests and ALPS-index** | | | | | | |  |  |
| --- | --- | --- | --- | --- | --- | --- | --- | --- |
|  |  | YOAD | YOAD | YOAD | CTL | CTL | CTL |  |
| Adjusted parameters |  |  | (edu) | (edu+age) |  | (edu) | (edu+age) |  |
| MMSE | ρ | 0.27^**^ | 0.25 ^**^ | 0.24^**^ | 0.01 | -0.03 | -0.05 |  |
|  | P-value | 1.10×10^-3^ | 1.85×10^-3^ | 2.70×10^-3^ | 0.44 | 0.65 | 0.71 |  |
| CASI Total (100) | ρ | 0.24^**^ | 0.22^**^ | 0.22^*^ | 0.11 | 0.06 | 0.02 |  |
|  | P-value | 4.63×10^-3^ | 9.88×10^-3^ | 0.01 | 0.13 | 0.27 | 0.43 |  |
| Mental Manipulation (10) | ρ | 0.14 | 0.11 | 0.11 | 0.22^*^ | 0.18^*^ | 0.17^*^ |  |
|  | P-value | 0.06 | 0.12 | 0.13 | 0.01 | 0.03 | 0.04 |  |
| Attention (8) | ρ | 0.13 | 0.11 | 0.11 | -0.04 | -0.07 | -0.08 |  |
|  | P-value | 0.09 | 0.12 | 0.13 | 0.65 | 0.77 | 0.79 |  |
| Orientation (18) | ρ | 0.18^*^ | 0.18^*^ | 0.17 | -0.03 | -0.03 | -0.05 |  |
|  | P-value | 0.03 | 0.03 | 0.03 | 0.64 | 0.63 | 0.71 |  |
| Long Term Memory (10) | ρ | 0.12 | 0.09 | 0.08 | 0.19^*^ | 0.16 | 0.13 |  |
|  | P-value | 0.11 | 0.18 | 0.19 | 0.02 | 0.05 | 0.08 |  |
| Short Term Memory (12) | ρ | 0.22^**^ | 0.21^*^ | 0.21^*^ | 0.21^*^ | 0.19^*^ | 0.16 |  |
|  | P-value | 9.32×10^-3^ | 0.01 | 0.01 | 0.01 | 0.03 | 0.05 |  |
| Abstract Thinking (12) | ρ | 0.10 | 0.06 | 0.06 | 4.70×10^-3^ | -0.02 | -0.03 |  |
|  | P-value | 0.14 | 0.25 | 0.26 | 0.48 | 0.59 | 0.61 |  |
| Drawing Ability (10) | ρ | 0.23^**^ | 0.21^*^ | 0.20^*^ | 0.08 | 0.07 | 0.06 |  |
|  | P-value | 7.03×10^-3^ | 0.01 | 0.02 | 0.20 | 0.23 | 0.28 |  |
| Verbal Fluency (10) | ρ | 0.17^*^ | 0.15 | 0.15 | -0.07 | -0.10 | -0.15 |  |
|  | P-value | 0.04 | 0.05 | 0.06 | 0.76 | 0.86 | 0.93 |  |
| Language (10) | ρ | 0.19^*^ | 0.17^*^ | 0.17^*^ | -0.12 | -0.13 | -0.13 |  |
|  | P-value | 0.02 | 0.04 | 0.04 | 0.89 | 0.91 | 0.90 |  |
| Data present as correlation coefficient, ^*^ p<0.05, ^**^ p<0.01, two-tailed  YOAD: Young onset Alzheimer’s disease; CTL: age-matched controls: ALPS: diffusion tensor imaging along the perivascular space  (edu): adjusted education years; (edu+age): adjusted education years and age at image  MMSE: Mini-Mental State Examination; CASI: Cognitive Ability Screening Instrument | | | | | | | | |

**eTable 3: Relationships between blood profiles and ALPS-index**

|  | CTL | | YOAD | |
| --- | --- | --- | --- | --- |
| Blood profiles | ρ | P-value | ρ | P-value |
| Homocysteine, μmole/L | 0.08 | 0.61 | -0.10 | 0.35 |
| Fasting Glucose | -0.31 | 0.07 | 3.87×10^-3^ | 0.97 |
| High sensitive C reactive protein (mg/L) | 0.20 | 0.17 | 0.01 | 0.93 |
| Hemoglobin A1C, % | -0.05 | 0.71 | 0.18 | 0.09 |
| Blood Urea Nitrogen, mg/dL | -0.09 | 0.63 | -0.05 | 0.68 |
| Calcium, mg/dL | 0.02 | 0.92 | -0.07 | 0.55 |
| Creatinine, mg/dL | 0.04 | 0.77 | -0.18 | 0.07 |
| Aspartate aminotransferase, U/L | -0.07 | 0.65 | -0.15 | 0.18 |
| Alanine aminotransferase, U/L | 0.01 | 0.94 | -0.04 | 0.71 |
| Total cholesterol, mg/dL | -0.22 | 0.12 | -5.44×10^-3^ | 0.96 |
| Triglyceride, mg/dL | -0.12 | 0.41 | -0.07 | 0.53 |
| High-density lipoprotein, mg/dL | -0.07 | 0.63 | 0.06 | 0.57 |
| Very low-density lipoprotein, mg/dL | -0.05 | 0.74 | -0.07 | 0.62 |
| Low-density lipoprotein, mg/dL | -0.16 | 0.25 | -0.05 | 0.65 |
| Cortisol, mcg/dL | 0.18 | 0.34 | -0.19 | 0.12 |
| T4, ng/dL | -0.37 | 0.05 | -0.15 | 0.28 |
| T3, ng/dL | 0.33 | 0.08 | 0.01 | 0.94 |
| TSH, μIU/mL | 0.09 | 0.63 | -0.05 | 0.70 |
| Vitamin B12, pg/dL | 0.05 | 0.75 | 0.03 | 0.77 |
| Folate, ng/dL | -0.06 | 0.69 | -0.04 | 0.70 |
| Albumin, g/dL | 0.03 | 0.93 | 0.13 | 0.70 |
| Hemoglobin, g/dL | 0.02 | 0.87 | -0.22 | 0.03 * |

^*^ P<0.05

YOAD: Young onset Alzheimer’s disease; CTL: age-matched controls; ALPS: diffusion tensor imaging along the perivascular space

**eTable 4: Gray matter Clusters showing atrophy in YOAD**

| Cluster | Anatomy & Lateralization | | X | Y | Z | T value | P value |
| --- | --- | --- | --- | --- | --- | --- | --- |
| I | Hippocampus | R | 34 | -24 | -14 | 12.04 | <0.0001 |
| II | Hippocampus | L | -33 | -24 | -12 | 10.39 | <0.0001 |
| III | Middle frontal gyrus | L | -27 | 36 | -12 | 6.8 | <0.0001 |
| IV | Superior frontal gyrus | R | 28 | -2 | 54 | 6.31 | <0.0001 |
|  | Frontal operculum | R | 44 | 12 | 32 | 6.27 | <0.0001 |
|  | Precentral gyrus | R | 50 | 8 | 39 | 5.76 | <0.0001 |
| V | Middle cingulate gyrus | R | 9 | 24 | 34 | 5.99 | <0.0001 |
|  | Anterior cingulate gyrus | R | 3 | 46 | 16 | 5.05 | 0.006 |
| VI | Superior-medial frontal gyrus | L | -12 | 38 | 18 | 5.48 | 0.001 |
| VII | Cerebellum crus | L | -28 | -66 | -36 | 5.09 | 0.005 |
|  | Cerebellum gray | L | -27 | -57 | -33 | 5.08 | 0.005 |

Atrophy regions compared with age-matched control, adjusted age at image and estimated total intracranial volume

YOAD: Young onset Alzheimer’s disease; R: right, L: left

X, Y, Z represent MNI coordinate; the anatomy is based on the AAL3 template

Family wise error P <0.05 for multiple comparison correction

**eTable 5: Gray matter clusters related to ALPS-index**

|  |  | Anatomy & lateralization | | X | Y | Z | T | P |
| --- | --- | --- | --- | --- | --- | --- | --- | --- |
| YOAD Cluster | K_E_ | Anatomy |  | X | Y | Z | T | P |
| I | 42330 | Amygdala-Hippocampus | R | 21 | 6 | -16 | 5.8 | <0.0001 |
|  |  | Superior frontal | L | -27 | 54 | -8 | 5.59 | <0.0001 |
|  |  | Thalamus | L | -20 | -30 | 3 | 5.49 | <0.0001 |
| II | 8024 | Cerebellum | R | 15 | -69 | -52 | 5.57 | <0.0001 |
|  |  | Cerebellum | L | -22 | -69 | -54 | 5.19 | <0.0001 |
| III | 1074 | Calcarine | R | 9 | -57 | 12 | 4.55 | <0.0001 |
| IV | 566 | Mid-frontal | L | -32 | 40 | 21 | 4.43 | <0.0001 |
|  |  | Superior frontal | L | -28 | 30 | 34 | 4.13 | <0.0001 |
| V | 101 | Supra-marginal | R | 56 | -21 | 26 | 3.87 | <0.0001 |
| VI | 100 | Mid-occipital | L | -34 | -69 | 36 | 3.81 | <0.0001 |
| VII | 228 | Mid-cingulate | L | -9 | -3 | 39 | 3.72 | <0.0001 |
| VIII | 129 | Sup-temporal | R | 66 | -8 | -8 | 3.7 | <0.0001 |
|  |  | Mid-temporal | R | 70 | -26 | -8 | 3.2 | 0.001 |
| IX | 128 | Inf-temporal | L | -54 | -46 | -16 | 3.67 | <0.0001 |
| X | 120 | Mid-cingulate | R | 9 | -50 | 34 | 3.67 | <0.0001 |
|  |  | Precuneus | R | 10 | -57 | 32 | 3.38 | <0.0001 |
| XI | 128 | Precuneus | L | -10 | -54 | 16 | 3.46 | <0.0001 |
| CTL Cluster | K_E_ |  |  |  |  |  |  |  |
| I | 9120 | Amygdala and hippocampus | R | 21 | 3 | -15 | 7.23 | <0.0001 |
|  |  | Thalamus | L | -15 | -32 | 3 | 5.11 | <0.0001 |
| II | 7644 | Amygdala and hippocampus | L | -22 | 2 | -15 | 7.2 | <0.0001 |
|  |  | Insula | L | -34 | -14 | 3 | 4.49 | <0.0001 |
| III | 1118 | Middle frontal | L | -32 | 42 | 21 | 5.86 | <0.0001 |
|  |  | Superior frontal | L | -16 | 62 | 15 | 4.53 | <0.0001 |
| IV | 340 | Middle frontal | R | 28 | 28 | 36 | 5.28 | <0.0001 |
| V | 1257 | Superior frontal | R | 27 | 57 | -9 | 4.9 | <0.0001 |
| VI | 3214 | Cerebellum gray | L | -34 | -51 | -44 | 4.93 | <0.0001 |
|  |  | Cerebellum crus | L | -30 | -78 | -39 | 4.7 | <0.0001 |
| VII | 3524 | Cerebellum crus | R | 24 | -81 | -36 | 4.85 | <0.0001 |
|  |  | Cerebellum gray | R | 28 | -70 | -44 | 4.66 | <0.0001 |
| VIII | 206 | Precentral | R | 24 | -14 | 57 | 4.64 | <0.0001 |
| IX | 466 | Inferior orbitofrontal | L | -42 | 39 | -9 | 3.19 | 0.001 |
| X | 254 | Inferior temporal | R | 48 | -50 | -26 | 3.94 | <0.0001 |
| XI | 179 | Sup-medial frontal | L | -10 | 48 | 3 | 3.92 | <0.0001 |

YOAD: Young onset Alzheimer’s disease; CTL: Age-matched controls; K_E:_ Cluster voxel numbers

R: right, L: left; ALPS: diffusion tensor imaging along the perivascular space

X, Y, Z represent MNI coordinate; the anatomy is based on the AAL3 template

P uncorrected, with cluster size >100 voxels

**eTable6:  Gray matter clusters related to mental manipulation and ALPS-index in controls**

| Cluster | Cluster  size | Anatomy & lateralization | | X | Y | Z | T | P |
| --- | --- | --- | --- | --- | --- | --- | --- | --- |
| I | 7560 | Amygdala | L | -18 | 0 | -15 | 7.00 | <0.0001 |
|  |  | Hippocampus | L | -28 | -10 | -12 | 6.27 | <0.0001 |
|  |  | Insula | L | -36 | -16 | 4 | 4.63 | <0.0001 |
| II | 8674 | Amygdala | R | 24 | 2 | -14 | 6.81 | <0.0001 |
|  |  | Thalamus Pulvinar | L | -8 | -24 | 9 | 5.44 | <0.0001 |
| III | 357 | Frontal Mid | R | 27 | 28 | 34 | 6.05 | <0.0001 |
| IV | 1426 | Frontal Mid | L | -32 | 40 | 20 | 5.90 | <0.0001 |
|  |  | Frontal Sup | L | -18 | 57 | 15 | 4.94 | <0.0001 |
| V | 4320 | Cerebellum Crus | R | 20 | -82 | -38 | 5.65 | <0.0001 |
|  |  | Cerebellum 7B | R | 30 | -70 | -45 | 4.81 | <0.0001 |
|  |  | Cerebellum 6 | R | 15 | -72 | -24 | 3.69 | <0.0001 |
| VI | 4093 | Cerebellum 8 | L | -34 | -54 | -44 | 4.93 | <0.0001 |
|  |  | Cerebellum Crus2 | L | -36 | -66 | -46 | 4.86 | <0.0001 |
| VII | 630 | Frontal Mid 2 | R | 34 | 46 | 9 | 4.79 | <0.0001 |
| VIII | 151 | Postcentral | R | 34 | -24 | 48 | 4.48 | <0.0001 |
| IX | 161 | Sub-genual | L | -14 | 48 | -15 | 4.29 | <0.0001 |
| X | 277 | Frontal Mid 2 | L | -33 | 48 | -9 | 4.18 | <0.0001 |
| XI | 132 | Olfactory | L | -2 | 10 | -12 | 3.90 | <0.0001 |
| XII | 261 | Temporal Inf | R | 48 | -56 | -24 | 3.84 | <0.0001 |
| XIII | 104 | Rectus | R | 6 | 36 | -22 | 3.66 | <0.0001 |
| XIV | 288 | Cuneus | R | 15 | -84 | 22 | 3.65 | <0.0001 |
|  |  | Occipital Mid | R | 39 | -74 | 28 | 3.65 | <0.0001 |

Data adjusted estimated total intracranial volume

R: right, L: left; ALPS: diffusion tensor imaging along the perivascular space

X, Y, Z represent MNI coordinate; the anatomy is based on the AAL3 template

P uncorrected, with cluster size >100 voxels

**eFigure Legends**

**eFigure 1**: Scatter plots between the ALPS-index and ages (A) and educational years (B) in control group (CTL) and in patients with young-onset Alzheimer’s disease (YOAD) (C: age, D educational years). ρ: Spearman’s ρ correlation, 95% C.I: 95% confidence interval, ALPS: diffusion tensor imaging along the perivascular space.

**eFigure 2**: Simple mediation diagram in patients with young onset Alzheimer’s disease; a, b, c, and c’ are path coefficients representing unstandardized regression weights and standard errors (in parentheses). The c path coefficient represents the total effect of the ALPS-index on short-term memory scores (STM). The c’ path coefficient refers to the direct effect of the ALPS-index on STM. All analyzed a, b, and c paths were significant, ^*^p < 0.05, ^***^p < 0.001. ALPS: diffusion tensor imaging along the perivascular space.

**eFigure 3**: Simple mediation diagram in controls; a, b, c, and c’ are path coefficients representing unstandardized regression weights and standard errors (in parentheses). The c path coefficient represents the total effect of the ALPS-index on mental manipulation scores (MM). The c’ path coefficient refers to the direct effect of the ALPS-index on MM. All analyzed a and c paths were significant, ^*^p < 0.05, ^**^p < 0.01, ^***^p < 0.001. ALPS: diffusion tensor imaging along the perivascular space. DLPFC: dorsolateral prefrontal cortex
