# Supplementary figures and images for: Gray matter reserve determines glymphatic system function in young‐onset Alzheimer's disease: Evidenced by DTI‐ALPS and compared with age‐matched controls
Source: Psychiatry Clin Neurosci. 2023 May 21;77(7):401–9. doi: 10.1111/pcn.13557 (PMC11488612; doi:10.1111/pcn.13557)

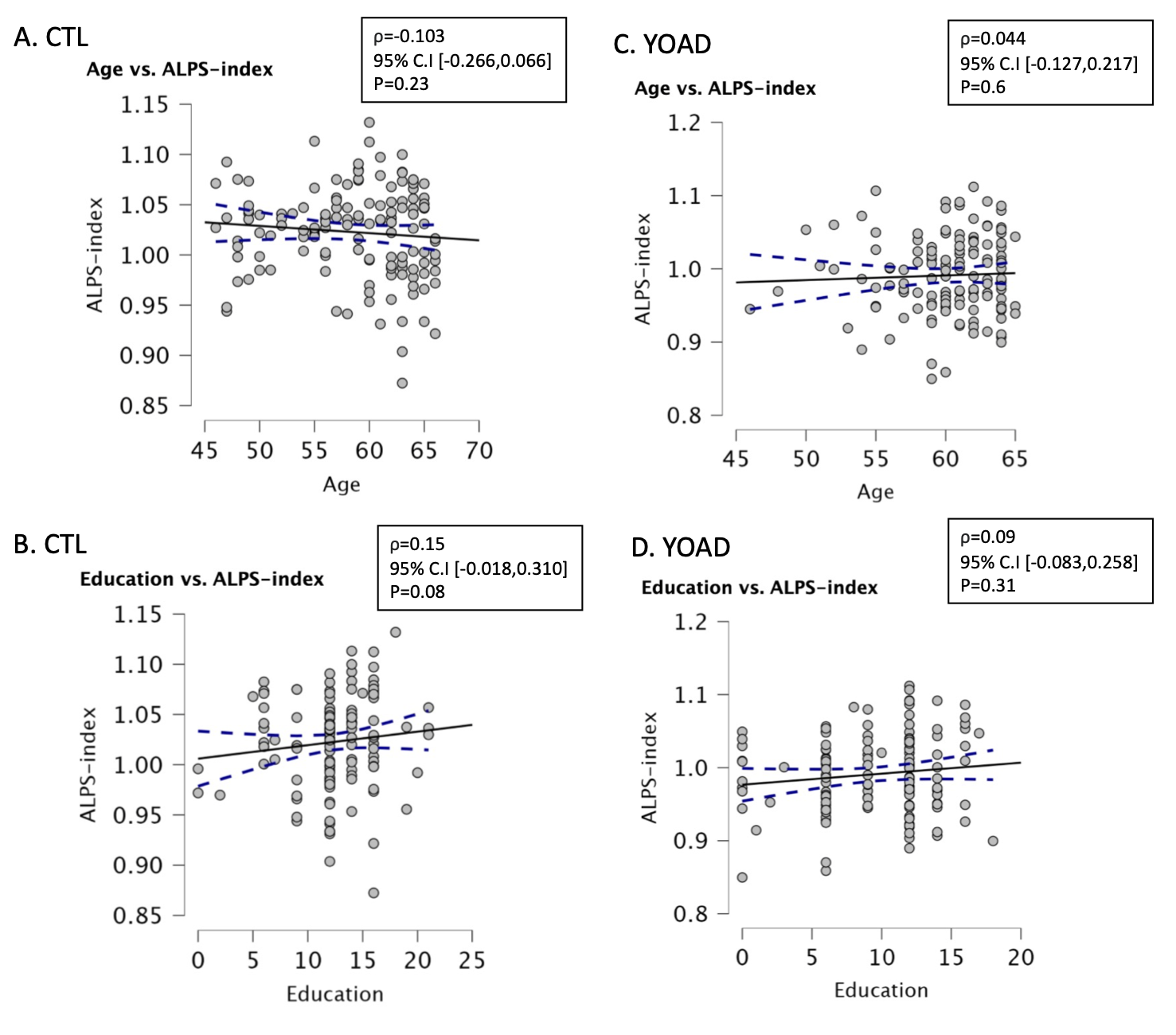

Supplement: Supplementary file 2 — Figure S1. Scatter plots between the ALPS‐index and ages (A) and educational years (B) in control group (CTL) and in patients with young‐onset Alzheimer's disease (YOAD) (C: age, D: educational years). [file PCN-77-401-s001.tiff]

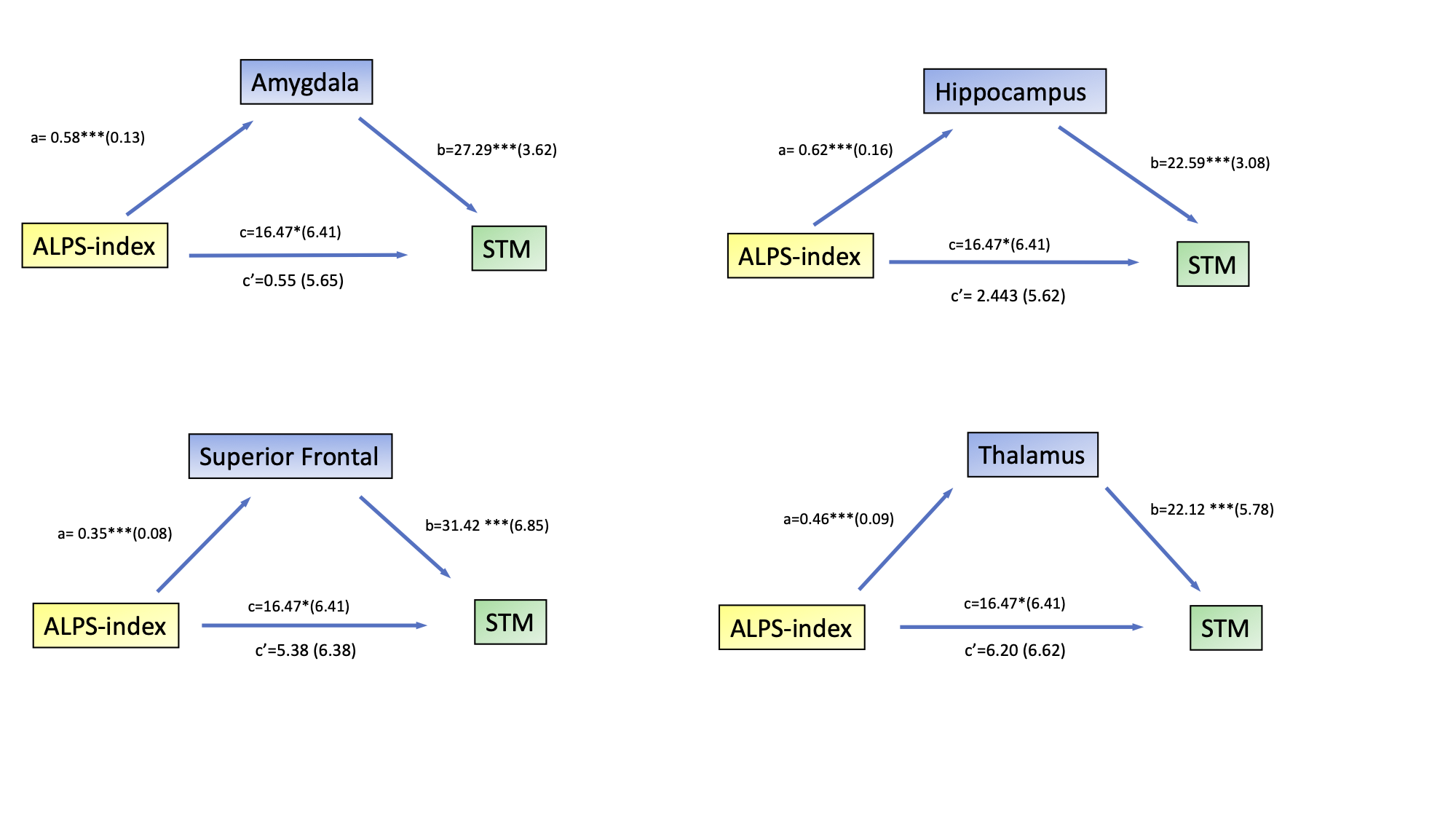

Supplement: Supplementary file 3 — Figure S2. Simple mediation diagram in patients with young onset Alzheimer's disease. [file PCN-77-401-s004.tiff]

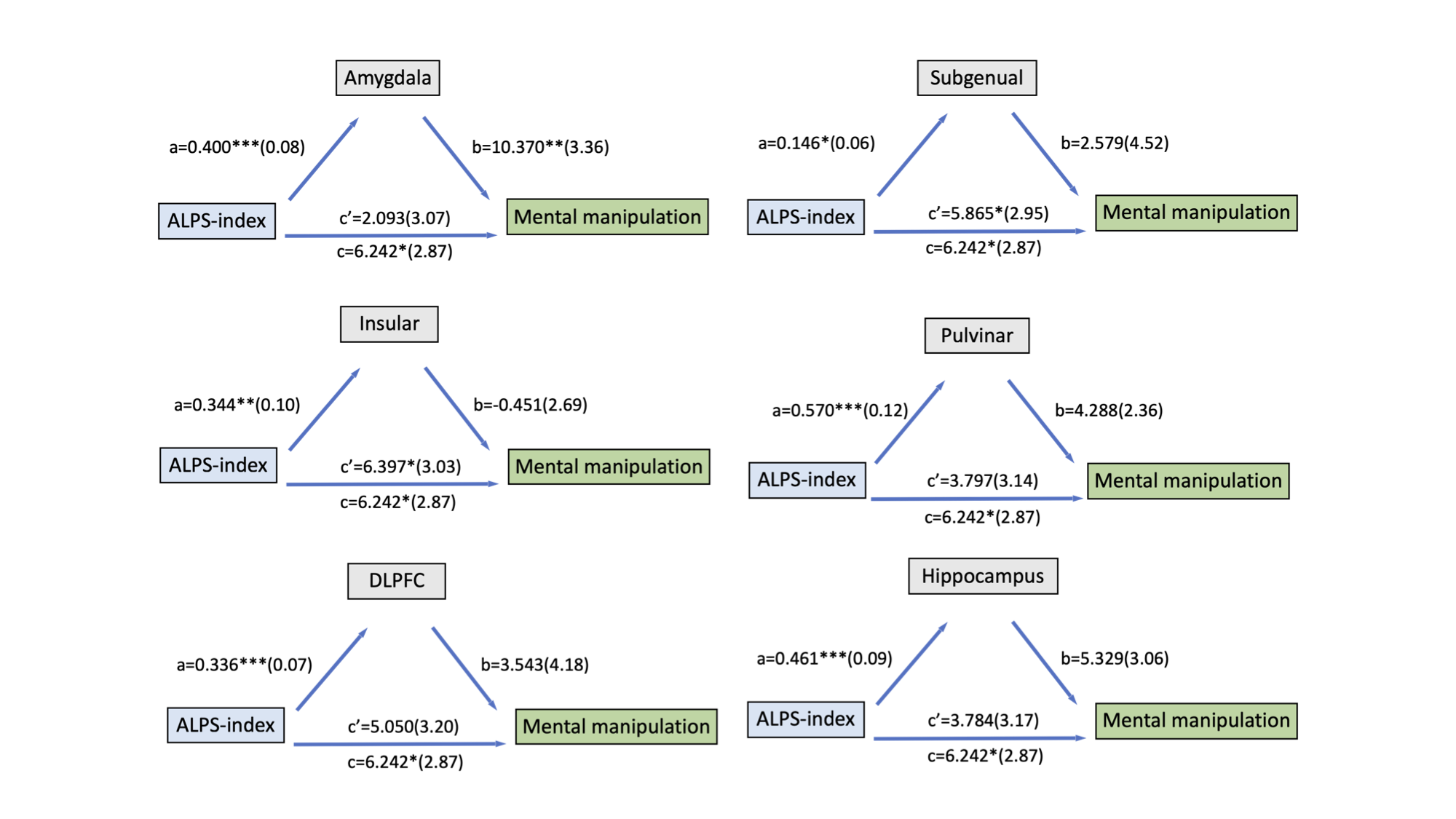

Supplement: Supplementary file 4 — Figure S3. Simple mediation diagram in controls. [file PCN-77-401-s002.tiff]
